# Sex-Specific Associations with Periodontal Inflammation and Bone Loss: A Cross-Sectional Analysis

**DOI:** 10.3390/dj14010011

**Published:** 2025-12-29

**Authors:** Valentin Bartha, Judith Schamuhn, Boris Krumm, Marco M. Herz

**Affiliations:** 1 Department for Conservative Dentistry, Heidelberg University, Im Neuenheimer Feld 400, 69120 Heidelberg, Germany; valentin.bartha@med.uni-heidelberg.de; 2 Private Practice, 50670 Cologne, Germany; judith_mohr@gmx.net; 3 Private Practice, 60439 Frankfurt, Germany; boris.krumm@gmx.de; 4 Department for Conservative Dentistry, University of Tübingen, Osianderstr. 2-8, 72076 Tuebingen, Germany

**Keywords:** sex, periodontitis, periodontal inflammation, periodontal pocket, sexual dimorphism

## Abstract

**Background**: To assess sex-related differences in periodontal inflammation and bone loss and identify sex-specific associations with systemic and local risk factors. **Methods:** This cross-sectional study analyzed records from a university setting. Outcomes were bleeding on probing (BOP) and bone loss index (BLI). Predictors included smoking, diabetes mellitus, age, plaque control record (PCR), the proportion of sites with pocket depth (PD) ≥ 5 mm, and number of teeth. Sex-stratified generalized linear models were applied. **Results:** A total of 232 participants were included (114 men, 118 women; mean age 55.6 ± 11.6 years). Men showed higher PD ≥ 5 mm (*p* = 0.030), with no sex difference in mean BOP or BLI. PD ≥ 5 mm predicted higher BOP in both sexes (men *p* < 0.001; women *p* = 0.002). Smoking was associated with higher BOP in men and with increased BLI in women (*p* = 0.010). PCR was positively associated with BOP in women and inversely with BLI in men (*p* = 0.042). **Conclusions:** In this study, sex-specific associations between behavioral/clinical factors and periodontal outcomes were observed. PD ≥ 5 mm was related to BOP in both sexes, while smoking and plaque control showed sex-divergent patterns. These exploratory findings warrant confirmation in prospective studies.

## 1. Introduction

The 21st century has already revealed itself to be an era of profound medical challenges.

At the dawn of the century, the complete sequencing of the human genome by the Human Genome Project (1990–2003) marked a fundamental paradigm shift: the advent of personalized medicine [1,2]. Building on genomic, molecular biological, and data-analytical insights, efforts have since been directed towards tailoring diagnostics and therapies to individual genetic, biological, and even lifestyle-related factors. What was initially discussed mainly within the field of oncology now shapes an ever-expanding range of medical disciplines. In parallel, gender medicine came increasingly into focus. It explores the interplay between biological sex and social or cultural dimensions of gender in health and disease. Early studies showed that women were long underrepresented in clinical trials and that medical standards often relied on male reference values. Today it is clear that cardiovascular and metabolic diseases, pain perception, and pharmacodynamics can differ markedly between women and men, affecting research, prevention, and healthcare. Growing evidence also shows sex-specific differences not only in disease risk but also in treatment response.

Personalized dentistry has emerged from the recognition that oral diseases do not manifest or progress in the same way for all individuals, and that therapeutic approaches should therefore be tailored accordingly. Building on advances in genomics, molecular diagnostics and digital technologies, biomarkers, genetic predispositions and risk profiles are increasingly being integrated into oral healthcare [3,4,5]. This enables more accurate predictions of individual risks for caries, periodontitis or peri-implant disease, as well as the development of patient-specific preventive and therapeutic strategies.

Oral diseases and their treatment outcomes differ not only between individuals but also systematically between women and men [6,7,8]. Early systematic reviews demonstrated sex-related differences in the prevalence and severity of destructive periodontal diseases, with men exhibiting higher rates of disease progression. More recent conceptual work has clarified the intersection of sex (biological differences) and gender (social and cultural determinants), showing that both dimensions significantly affect susceptibility, clinical presentation, and therapeutic responses in oral health. Sex-related differences are particularly evident in periodontitis. Men show higher prevalence and severity, with ~9% more destructive cases than women [8,9]. Animal models demonstrate greater bone loss in males under equal challenge, linked to heightened neutrophil activity and osteoclast precursor recruitment [10]. Human studies confirm sex-specific interactions between systemic health factors and periodontal inflammation [11]. Oral microbiome profiles also differ: men harbor more spirochetes, women more Synergistota and Firmicutes—summarized as the “microsexome” [12]. Hormonal fluctuations further modulate female periodontal health: gingivitis during puberty, menstruation, and pregnancy reflects hormone-driven vascular and immune changes [13,14,15], while postmenopausal estrogen decline increases susceptibility to bone loss and tissue destruction [16]. Estrogen may also influence *P. gingivalis* virulence by altering cytokine expression and biofilm pathogenicity [4,17]. Behavioral factors further contribute to sex differences in oral health. Men generally exhibit poorer oral hygiene, attend dental check-ups less frequently, and have higher rates of periodontal disease and oral cancer (a nearly 2:1 ratio) [16,18,19]. Smoking disproportionately affects men, amplifying periodontal risk [12].

The clinical patterns by which sex affects periodontal inflammation and tissue destruction are still not well characterized. Few studies have assessed whether such differences extend to structural outcomes like alveolar bone loss. Population-based analyses are needed to clarify how biological sex affects both inflammatory and destructive components of periodontitis. Some researchers pooled the patient groups investigated in their previously published studies and subjected them to a secondary analysis in order to better address these research questions [11,12].

Based on the sex-specific patterns reported in previous clinical and experimental studies, we hypothesized that men and women may differ not only in periodontal inflammation (BOP) but also in age-adjusted radiographic bone loss, and that systemic and local risk factors may exhibit sex-specific associations. Therefore, we re-evaluated patients with periodontitis to reassess sex-related differences in disease manifestation, pooling population-based cohorts from several of our previous investigations. The primary objective was to compare clinical inflammation, measured as bleeding on probing (BOP), between men and women. Secondary objectives were to analyze sex-specific associations of systemic factors (smoking, diabetes) and local factors (plaque levels, probing depth, number of teeth) with BOP and radiographically assessed bone loss.

## 2. Methods

Study design and participants

This cross-sectional analysis re-examined baseline data from patients of the Tübingen TüPaRetro-studies who received periodontal treatment at the University Dental Clinic between 2004 and 2016 [20,21,22]. The protocol was approved by the local ethics committee (557/2016BO2); procedures complied with the Declaration of Helsinki and STROBE guidelines [23]. Furthermore, the analytic plan was pre-specified and guided by Farina et al. to enhance methodological comparability [11].

### 2.1. Eligibility Criteria

#### 2.1.1. Inclusion Criteria

**Figure 1:**
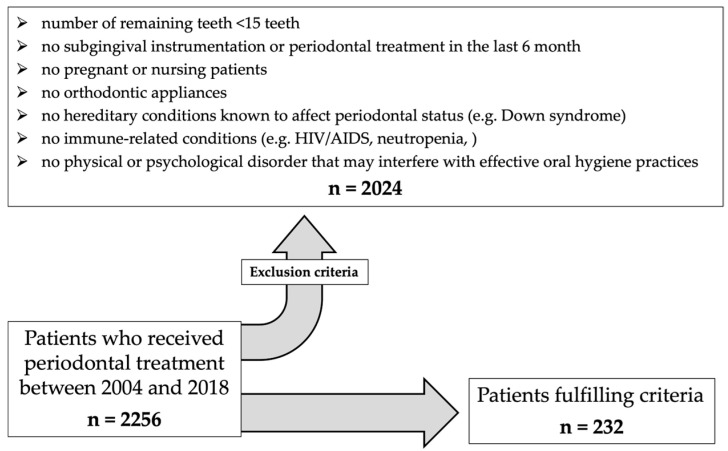
Process of patient selection.

Participants were included in the study if they met all of the following criteria:

➢ Initial examination at baseline
  ○ Participants age ≥18 years
  ○ Diagnosis of periodontitis Stage II bis IV
➢ Complete dental and periodontal examination
➢ Radiographic examination ≤ 12 months prior to clinical examination
➢ Smoking status (non-smoker, former smoker (≥5 years), smoker)
➢ Detailed medical history focusing on specific diseases or disorders (Figure 1)

#### 2.1.2. Exclusion Criteria

The exclusion criteria encompassed a wide range of aspects and parameters. An overview of the numerous exclusion criteria is provided in Figure 1.

### 2.2. Clinical Data Collection

Periodontal pocket depth (PD) was measured at six sites per tooth; bleeding on probing (BOP) was recorded per site and expressed as % of sites per patient. Plaque control record (PCR) was assessed [24].

All clinical examinations were conducted by general dentists or periodontists and, if conducted by undergraduates, subsequently subjected to verification by either dentists or periodontists. The recording of dental and periodontal parameters followed a strict standardized protocol. Fixed forms, a precisely defined examination and tooth sequence, and identical diagnostic instruments were used throughout the entire study period. All examinations were performed after prior supragingival cleaning to minimize difficulties in measuring and reading the values. Bleeding on probing (BOP) was recorded on a site-specific level as present or absent (yes/no) following the respective probing depth measurement. On the patient level, BOP was expressed as the percentage (%) of all probed sites in the full dentition (BOP%). In addition, the number of teeth and the percentage (%) of all sites with a PD ≥ 5 mm was evaluated.

### 2.3. Radiographic and Demographic Data

When periapical radiographs are used for calculations, they are always taken using a standardized protocol with the right-angle technique, with the patient seated upright and using a bite block. For panoramic radiographs, patients likewise assume the same defined upright position with a bite block, following standard instructions.

Alveolar bone loss was expressed as a bone loss index (BLI = maximum bone loss per age). For each tooth, the greatest CEJ–bone level measurement at either the mesial or distal site was recorded. The BLI was calculated using the highest value across all teeth, representing the patient’s worst bone loss site. This approach provides a single, conservative estimate of disease severity and allows for easier comparison between individuals [25,26].

Demographic variables included age, sex, smoking status (current, former, non-smoker), and diabetes mellitus. HbA1c values and smoking intensity (e.g., pack-years) were unavailable.

### 2.4. Bias Considerations and Data Quality

Selection bias was possible due to the exclusion of incomplete records; information bias could arise from self-reported smoking status. To minimize these limitations, only records with complete, verifiable data on PD, BOP, BLI, and systemic health variables were included.

### 2.5. Sample Size and Post Hoc Power Analysis

No formal sample size calculation was performed due to the retrospective design. All eligible participants were included (n = 232). Post hoc assessment indicated that the study had limited power to detect small sex-related differences in BOP.

### 2.6. Statistical Analysis

Statistical analyses were performed using JMP Pro 17.0 (SAS Institute Inc., Cary, NC, USA). Continuous variables were assessed for normality using the Anderson Darling Test and quantile plots. Group differences between men and women were assessed using independent *t*-tests or Wilcoxon rank-sum tests, as appropriate.

Categorical variables were compared using chi-squared or Fisher’s exact tests. Multivariable regression analyses using generalized linear models (Gamma distribution with loglink) were conducted separately for men and women, including independent variables such as smoking status, diabetes mellitus, age, PCR (%), number of teeth, and the proportion of sites with PD ≥ 5 mm. Significance was set at *p* < 0.05.

Graphical visualization of the associations between BOP (%), Bone loss index and selected predictors (smoking status, PD ≥ 5 mm and PCR) was performed using automated effect plots in JMP Pro 17.0. These plots represent linear regression-based estimations with 95% confidence intervals and serve illustrative purposes. Due to software constraints, confidence bands are not derived from the final generalized linear model (Gamma distribution with log-link) but provide a visual approximation of the direction and sex-specific strength of associations.

Generative artificial intelligence (ChatGPT 5.0, OpenAI) was used in this manuscript to refine prewritten sections in accordance with journal guidelines, including reducing word count and overall length, ensuring consistency, and improving language editing.

## 3. Results

A total of 232 participants were included, of whom 114 were men and 118 were women.

The age range was from 20 to 82 years (mean age: 55.6 ± 11.6 years). In total, there were 57 (24.6%) smokers and 20 (8.6%) participants with diabetes mellitus. A detailed overview of the baseline patient characteristics is provided in Table 1.

### 3.1. Factors Associated with BOP (%) in Females and Males

**Figure 2:**
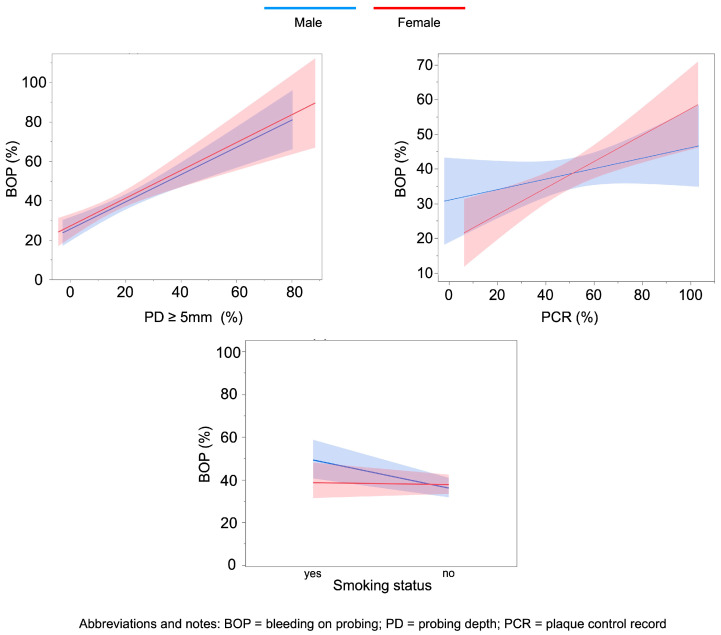
Illustrative sex-stratified associations between BOP% and selected predictors: proportion of sites with PD ≥ 5 mm, PCR%, and smoking status. Lines represent linear regression-based estimations with 95% confidence intervals, generated for visualization purposes and not directly derived from the final generalized linear model (Gamma distribution, log link).

Sex-stratified generalized linear models (Gamma distribution, log link) assessed associations of smoking status, diabetes mellitus, age, PD ≥ 5 mm, number of teeth, and plaque control record (PCR) with BOP (%) (Figure 2; Table 2).

In both sexes, the proportion of sites with PD ≥ 5 mm was the strongest predictor (men: β = 1.52, 95%CI:0.86–2.19, *p* < 0.001; women: β = 1.39, 95%CI:0.53–2.25, *p* = 0.002).

In men, smoking was positively associated with BOP (β = 0.16, 95%CI:0.02–0.31, *p* = 0.029), whereas no such effect was found in women. Conversely, PCR was significantly associated with BOP in women (β = 0.009, 95%CI:0.003–0.015, *p* = 0.003), but not in men. Other covariates (diabetes mellitus, age, and number of teeth) showed no significant associations in either sex.

### 3.2. Factors Associated with Bone Loss Index

**Figure 3:**
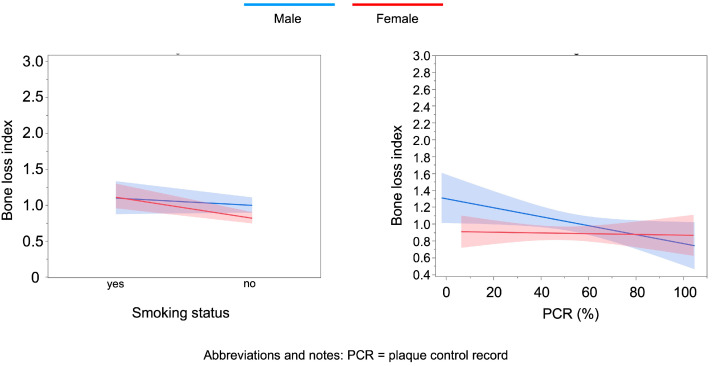
Illustrative sex-stratified associations between bone loss index and selected predictors: smoking status and PCR%. Lines represent linear regression-based estimations with 95% confidence intervals, generated for visualization purposes and not directly derived from the final generalized linear model (Gamma distribution, log link).

Sex-stratified generalized linear models (Gamma distribution, log link) were used with bone loss index (maximum bone loss per age) as the outcome (Table 3; Figure 3). In men, PCR showed a significant inverse association with age-adjusted bone loss. In women, smoking was the only significant predictor, being positively associated with greater bone loss.

## 4. Discussion

We found sex-specific differences in periodontal disease presentation and associated risk factors. While overall BOP did not differ between sexes, men had a higher proportion of sites with PD ≥ 5 mm. In both sexes, PD ≥ 5 mm was the strongest predictor of BOP, but smoking was associated with higher BOP only in men, whereas plaque levels (PCR) were positively associated with BOP only in women. For bone loss, smoking was the only significant risk factor in women, while PCR showed an inverse association with age-adjusted bone loss in men. These findings indicate that behavioral and clinical risk factors affect periodontal inflammation and tissue destruction differently in men and women, supporting the need for sex-specific approaches in diagnosis and prevention.

Biological sex influences periodontal inflammation, BOP, and alveolar bone loss through immunological, hormonal, microbiological, and behavioral pathways. A systematic review by Shiau & Reynolds reported consistently more severe periodontal disease in men, with deeper pockets and higher BOP [8]. While our findings align with their results for PD, we did not observe sex differences in BOP. Differences in study populations may explain this, as Shiau & Reynolds noted substantial heterogeneity in diagnostic criteria, and our cohort was more homogeneous.

Farina et al. similarly found higher BOP in men [11]; the discrepancy with our results may also reflect unmeasured lifestyle factors such as diet, which can modulate the plaque–inflammation relationship [27,28]. Furuta et al. showed that poorer hygiene in males can be compounded by greater inflammatory reactivity [29]. Consistent with Farina et al. we found PD ≥ 5 mm to be the strongest predictor of BOP in both sexes, with subtle differences in effect size suggesting biological or behavioral modulation [11]. These observations highlight the value of sex-stratified analyses in periodontal research.

The observed positive association between smoking and BOP in men contrasts with the widely reported anti-inflammatory effects of tobacco use, often attributed to nicotine-induced vasoconstriction and immunosuppression [11,30]. However, our dataset did not consistently capture smoking intensity. It is therefore possible that a substantial proportion of participants were light or occasional smokers, in whom vasoconstrictive effects may be less pronounced. In such cases, the pro-inflammatory impact of smoking could dominate, leading to increased clinical signs of inflammation, as reflected by BOP.

In this study, smoking was associated with a slightly increased bone loss in women, but not in men, which stands in contrast to the common assumption—supported by several other studies—of a stronger effect in men [31,32,33,34,35]. Despite the lack of precise information on the number of cigarettes smoked—which calls for cautious interpretation of the association between women and smoking—it should be noted that female smokers in this study still showed a tendency toward poorer bone levels, a finding that was statistically significant.

Our dataset cannot test mechanistic pathways; however, in the context of prior research, the lack of association in men may reflect lower variance in exposure or biological resistance, while the predominantly postmenopausal status of many women could have amplified bone loss through combined hormonal and toxic effects [36]. No age-related effects were observed in either sex.

Although age-related effects on periodontal health have been reported, we did not observe those effects in our population [37]. Particularly regarding BOP, the mechanisms in relation to age and sex might be complex, as age reduces vascularization, which may affect bleeding. The inhomogenic age distribution of our cohort might have masked such effects globally.

In a Swiss cross-sectional study, the proportion of individuals who “claimed periodontitis” was also higher among women (53.9% vs. 49.1%), which the authors interpret as a selection or utilization effect, since women tend to seek dental care more frequently [38].

Johnson & Wikle showed that male gingival tissues express higher levels of pro-inflammatory cytokines (IL-1β, TNF-α), promoting connective tissue degradation and leukocyte recruitment, whereas female tissues display greater apoptotic and anti-inflammatory activity, suggesting enhanced immune resolution [39]. These mechanisms are supported by previous experimental work but cannot be inferred directly from our cross-sectional data and should therefore be viewed as hypotheses rather than causal explanations. They may underlie the higher proportion of sites with PD ≥ 5 mm in men in our cohort. Supporting evidence from animal models shows that male mice exhibit more severe bone loss, increased RANKL expression, and greater neutrophil infiltration following periodontal infection [40]. Furthermore, these males had elevated neutrophil infiltration, supporting the hypothesis that sex influences both immune activation and bone resorption pathways. Sangalli et al. emphasized the regulatory effects of estrogen, noting that this hormone can limit inflammatory cytokine production and enhance connective tissue integrity, providing a potential explanation for the relatively milder disease in females [9]. Similar sex-linked immune modulation was observed in obese SS LepR mutant rats [41].

In our study, bone loss index was higher in men, though not statistically significant (*p* = 0.06). Beyond these overall differences in bone loss, our sex-stratified models revealed an inverse association between PCR and age-adjusted bone loss in men. Men generally exhibit greater variability in oral-hygiene behavior and health-related routines, which may weaken the correspondence between current plaque levels and cumulative tissue destruction [18,19]. In contrast, women typically show more consistent health and oral-hygiene behaviours, which may lead to a closer alignment between present plaque status and periodontal outcomes [18,19]. These sex-specific behavioral patterns could therefore contribute to the inverse PCR–BLI association observed in men and highlight the importance of incorporating lifestyle factors in future sex-stratified analyses. However, this interpretation remains hypothetical and cannot be confirmed within the present dataset. Microbiome differences may also contribute: men harbor more virulent pathogens (e.g., *P. gingivalis, T. denticola, T. forsythia*), whereas women show higher prevalence of health-associated species such as *S. sanguinis* [12]. Animal studies further confirm sex-specific host–pathogen interactions, with males showing stronger neutrophilic responses and higher pro-inflammatory markers, and females maintaining lower bacterial colonization and better epithelial barrier integrity [42].

In line with Farina et al., we found no sex-related differences in the impact of diabetes mellitus on periodontal inflammation or bone loss [11]. Interpretation is limited by missing HbA1c data, preventing assessment of glycemic control. In contrast, Liu et al. reported stronger associations between type 2 diabetes and periodontal breakdown in men, potentially due to poorer glycemic control, reduced inflammatory resolution, or less effective oral hygiene [43]. These findings are consistent with broader evidence that men engage less in preventive dental care, delay visits, and have higher tobacco use, all contributing to worse periodontal outcomes [18].

Overall, our results indicate that biological sex could be a critical determinant in the pathogenesis and management of periodontal diseases. Men tend to exhibit greater inflammatory reactivity, deeper pockets, higher BOP, and more severe bone loss. In the context of previous research, these observations could be driven by behavioral risks, microbiome composition, hormonal influences, and innate immune responses. As our dataset cannot test these mechanisms directly, they should be interpreted as literature-based hypotheses rather than causal explanations. Future studies should integrate mechanistic, longitudinal, and sex-stratified designs to clarify which of these pathways most strongly contribute to the observed clinical patterns.

### Limitations and Strengths

This study has several limitations that should be addressed:

(1) The retrospective design represents a methodological limitation, as the analysis was confined to pre-existing documentation. Consequently, relevant information may be missing either due to a lack of perceived importance at the time of data collection or due to unintentional omissions in the clinical records.
(2) One of these missing and at that time not collected data points concerns the HbA1c value, which was recorded very inadequately and is still hardly ever known by the patient today.
(3) Another data gap concerns the exact smoking behaviour. Although it was recorded whether the patient smokes, quit smoking (for at least 5 years), or is a non-smoker, the precise number of cigarettes or packs per day was not asked during the medical history interview at that time. The amount of cigarettes actually consumed certainly has a not insignificant influence.
(4) The patient examinations were conducted by uncalibrated examiners. However, the recording of dental and periodontal parameters adhered to a very strict protocol.
(5) The timing within the female menstrual cycle was not recorded (potential for additional hormonal influence); however, the median age of 57 among female participants suggests a predominantly postmenopausal cohort.
(6) Potentially relevant dietary and lifestyle factors influencing periodontal inflammation were not assessed in this study.
(7) It should also be noted that the bacterial flora of the patients was not examined; therefore, patients with particularly aggressive periodontal pathogens could not be selected.

This university-based cohort may not fully represent the general population, and the inclusion of only complete records could limit external validity. However, this standard retrospective approach strengthens internal validity, and future population-based prospective studies are needed to confirm generalizability.

Strengths of this study include its focus on a clinically relevant and timely research question, as sex-specific analyses are an emerging priority in dentistry. The relatively large sample size after strict exclusions and the near-equal distribution of male and female participants enhance the robustness of the findings.

The observed sex-specific associations in this study underscore the potential value of integrating systemic and sex-based risk profiles into periodontal diagnostics and prevention. Such an approach may help to better tailor interventions, but prospective research is required to determine its clinical utility. Moreover, both our findings and the supporting literature underscore that sex differences likely amplify the clinical relevance of systemic factors for local periodontal inflammation.

## 5. Conclusions

Acknowledging the constraints of the retrospective study design, our analysis revealed sex-specific patterns in periodontal disease manifestation. Probing depth was the strongest predictor of bleeding on probing in both sexes, while smoking was associated only in men and plaque control only in women. For bone loss, smoking showed an association in women—although this finding should be interpreted with caution, as detailed information on the exact number of cigarettes consumed (e.g., pack-years) was not available—whereas better plaque control was linked to lower values in men. These findings suggest that sex may influence the relationship between behavioral or clinical factors and periodontal outcomes. Prospective studies with standardized data collection are needed to confirm these associations and clarify their underlying mechanisms.

Taken together, our findings highlight the continued importance of integrating personalized and sex-specific perspectives into dental research and clinical practice, encompassing both the investigation of underlying causes and the development of therapeutic interventions.

## Figures and Tables

**Table 1:** Baseline characteristics.

Parameter	Total (Mean ± SD)	Men (Mean ± SD)	Women (Mean ± SD)	*p*-Value (Male vs. Female)
Age (years)	55.6 ± 11.6	55.4 ± 11.8	55.8 ± 11.5	0.758
PD ≥ 5 mm (%)	17.4 ± 14.8	19.6 ± 15.2	15.3 ± 14.2	0.030 *
BOP (%)	38.4 ± 23.4	39.0 ± 23.5	37.9 ± 23.5	0.717
PCR (%)	51.0 ± 20.2	52.6 ± 20.1	49.5 ± 20.2	0.072
Bone loss index	0.95 ± 0.51	1.01 ± 0.51	0.89 ± 0.51	0.060
Smoking (yes)	24.6%	22.8%	26.3%	0.547
Diabetes (yes)	8.6%	9.6%	7.6%	0.645

Abbreviations and notes: PD = pocket depth; PCR = plaque control record; * = statistically significant.

**Table 2:** Multivariable generalized linear models (Gamma, log-link) predicting BOP% in men and women. Estimates, 95%CI, and *p*-values are reported; age ≥ 70 years serves as the reference. Significant predictors (*p* < 0.05) are marked with an asterisk.

Predictor		Men: Estimate (95% CI)	*p*-Value	Women: Estimate (95% CI)	*p*-Value
Smoking status		0.16 (0.02; 0.31)	0.029 *	–0.03 (–0.16; 0.09)	0.587
Diabetes mellitus		–0.07 (–0.26; 0.12)	0.471	0.13 (–0.07; 0.33)	0.194
PD ≥ 5 mm (%)		1.52 (0.86; 2.19)	<0.001 *	1.39 (0.53; 2.25)	0.002 *
Age (years)	20–29	–0.21 (–1.21; 0.80)	0.683	0.35 (–0.17; 0.87)	0.186
	30–39	0.09 (–0.32; 0.49)	0.667	0.06 (–0.32; 0.45)	0.739
	40–49	0.05 (–0.26; 0.36)	0.756	–0.13 (–0.39; 0.12)	0.305
	50–59	–0.11 (–0.41; 0.18)	0.453	–0.09 (–0.31; 0.13)	0.419
	60–69	0.10 (–0.22; 0.42)	0.526	–0.08 (–0.30; 0.14)	0.473
Ref. Age ≥ 70		–	–	–	–
Number of teeth		–0.002 (–0.03; 0.03)	0.912	0.03 (–0.01; 0.06)	0.128
PCR (%)		0.005 (–0.0004; 0.01)	0.071	0.009 (0.003; 0.015)	0.003 *

Abbreviations and notes: PD = pocket depth; PCR = plaque control record; CI = confidence interval; * = statistically significant.

**Table 3:** Multivariable models predicting the bone loss index in men and women. Estimates.

Predictor	Men: Estimate (95% CI)	*p*-Value	Women: Estimate (95% CI)	*p*-Value
Smoking status	0.06 (–0.12; 0.25)	0.483	0.13 (0.03; 0.21)	0.010 *
Diabetes mellitus	–0.07 (–0.30; 0.17)	0.564	–0.01 (–0.19; 0.17)	0.912
Number of teeth	–0.03 (–0.08; 0.02)	0.288	–0.01 (–0.05; 0.03)	0.545
PCR (%)	–0.008 (–0.016; –0.0002)	0.042 *	0.0002 (–0.0045; 0.0049)	0.941

Abbreviations and notes: PCR = plaque control record; CI = confidence interval; bone loss index = maximum bone loss per age; * = statistically significant.

## Data Availability

The datasets used and/or analyzed during the current study are available from the corresponding author on reasonable request.

## Abbreviations

The following abbreviations are used in this manuscript:

ADHA	Attention-Deficit/Hyperactivity Disorder
BLI	Bone loss Index
BOP	Bleeding on probing
CEJ	Cementoenamel junction
CI	Confidence interval
HbA1c	glycated hemoglobin (hemoglobin A1c)
IL-1β	Interleukin-1 beta
LDL	low-density protein
PCR	Plaque control record
HIV	Human Immunodeficiency Virus
AIDS	Acquired Immunodeficiency Syndrome
PD	pocket depth
RANKL	Receptor Activator of Nuclear Factor kappa-B Ligand
SD	Standard deviation
SS LepR	Dahl salt-sensitive (rat strain), Leptin receptor
TNF-α	Tumor Necrosis Factor alpha
